# Supplementary material for: Drug repurposing for aging research using model organisms
Source: Aging Cell. 2017 Jun 16;16(5):1006–15. doi: 10.1111/acel.12626 (PMC5595691; doi:10.1111/acel.12626)
Supplement: Supplementary file 7 — Data S1 Zip‐Archive of all report cards. [file ACEL-16-1006-s007.zip › RC_AMP.pdf]

# AMP

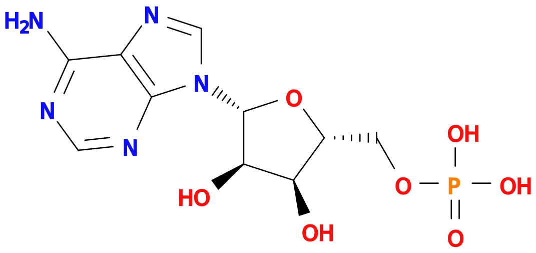


**Database identifiers**

| | |
|---|---|
| ChEMBLCompound | CHEMBL752 |
| DrugBank | DB00131 |
| CHEBI | 16027 |
| eMolecules | 711281 |

## Ranking

| | Rank | Score |
|---|---|---|
| Drosophila | 292/697 | 0.569 |
| C. elegans | 386/591 | 0.149 |

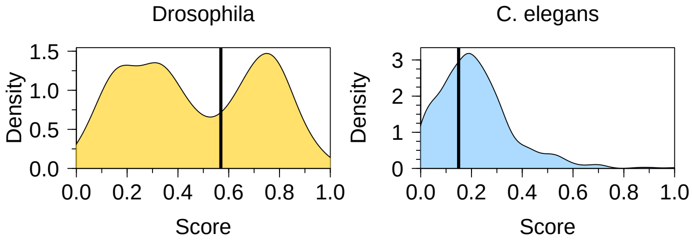


| | Ageing implication | Domain conservation | Binding site conservation | Binding affinity | Bioavailability | Lipinski | Promiscuity | Purchasability | Drug approval | **Total** |
|---|---|---|---|---|---|---|---|---|---|---|
| Drosophila | 1.0 | 0.974 | 1.0 | 0.62 | (0.9) | -0.05 | -0.1 | 0.1 | 0.075 | 0.569 |
| C. elegans | 1.0 | 0.972 | 1.0 | 0.62 | 0.206 | -0.05 | -0.1 | 0.1 | 0.075 | 0.149 |

## Names

- **AMP**
- 5'-O-phosphonoadenosine
- A-5MP
- adenosine 5'-(dihydrogen phosphate)
- Adenosine 5'-phosphate
- Adenosine Monophosphate
- Adenosine-5'-monophosphoric acid
- adenosine-5'P
- Adenyl
- Adenylate
- Adenylic acid
- Ado5'P
- My-B-Den
- pA
- PAdo
- Vitamin B8

## Roles

micronutrient, nutraceutical, fundamental metabolite, EC 3.1.3.11 (fructose-bisphosphatase) inhibitor, EC 3.1.3.1 (alkaline phosphatase) inhibitor, adenosine A1 receptor agonist

## Status

| | |
|---|---|
| Approved drug (according to ChEMBL) | No |
| Classification (according to DrugBank) | approved, nutraceutical |
| Number of Rule of 5 violations | 1 |
| Binding affinity to original target in log units (RF-Score prediction) | 5.49 |
| Burns *C. elegans* bioavailability prediction | -6.93 |

## Medical Information

**Indicatation:** For nutritional supplementation, also for treating dietary shortage or imbalance
**Mechanism of action:** Nucleotides such as Adenosine-5'-Monophosphate affect a number of immune functions, including the reversal of malnutrition and starvation-induced immunosuppression, the enhancement of T-cell maturation and function, the enhancement of natural killer cell activity, the improvement of delayed cutaneous hypersensitivity, helping resistance to such infectious agents as Staphylococcus aureus and Candida albicans, and finally the modulation of T-cell responses toward type 1 CD4 helper lymphocytes or Th1 cells. Studies have shown that mice fed a nucleotide-free diet have both impaired humoral and cellular immune responses. The addition of dietary nucleotides normalizes both types of responses. RNA, a delivery form of nucleotides, and ribonucleotides were used in these studies. The mechanism of the immune-enhancing activity of nucleic acids/nucleotides is not clear.
**Retail:** Spectrum Pharmaceuticals
Adenosine-5 monophosphate - 19.74 USD / g

(Information from DrugBank)

## Compound Target Characteristics

### Proto-oncogene tyrosine-protein kinase receptor Ret

Best gene implication in ageing for this target family came from gene P07949 via mapping the annotation from Ensembl ENSG00000165731 via mapping the annotation from EntrezGene 5979 via mapping the annotation from GenAgeHuman 0056 annotated in GenAge release 17.

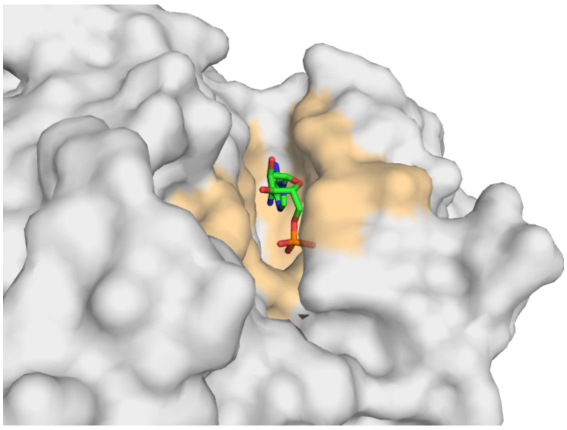

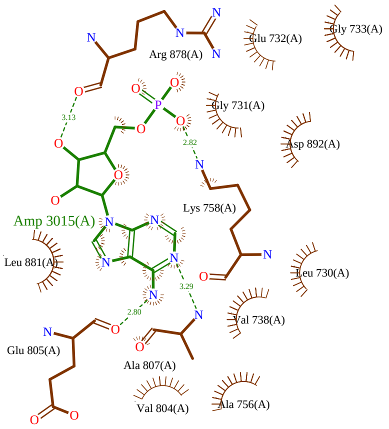

| protein | amino acids contacts (binding site) | | | | | | | | | | | | | |
|---|---|---|---|---|---|---|---|---|---|---|---|---|---|---|
| PDB:2ivt:chainA:P07949 | L | G | E | G | V | A | K | V | E | A | R | N | L | D |
| tr:B4DGX8:B4DGX8_HUMAN | L | G | E | G | V | A | K | V | E | A | R | N | L | D |
| sp:P07949:RET_HUMAN | L | G | E | G | V | A | K | V | E | A | R | N | L | D |
| sp:G3V9H8:RET_RAT | L | G | E | G | V | A | K | V | E | A | R | N | L | D |
| sp:P35546:RET_MOUSE | L | G | E | G | V | A | K | V | E | A | R | N | L | D |
| tr:Q7JQD5:Q7JQD5_DROME | L | G | E | G | V | A | K | I | E | A | R | N | L | D |
| tr:Q7KA88:Q7KA88_DROME | L | G | E | G | V | A | K | I | E | A | R | N | L | D |
| tr:Q7KT06:Q7KT06_DROME | L | G | E | G | V | A | K | I | E | A | R | N | L | D |
| tr:Q9BHA9:Q9BHA9_DROME | L | G | E | G | V | A | K | I | E | A | R | N | L | D |

| | whole protein | | domain-based | | contact-based | |
|---|---|---|---|---|---|---|
| protein | ident | simil | ident | simil | ident | simil |
| PDB:2ivt:chainA:P07949 | 0.99 | 1.0 | 1.0 | 1.0 | 1.0 | 1.0 |
| tr:B4DGX8:B4DGX8_HUMAN | 0.73 | 0.73 | 1.0 | 1.0 | 1.0 | 1.0 |
| sp:P07949:RET_HUMAN | 1.0 | 1.0 | 1.0 | 1.0 | 1.0 | 1.0 |
| sp:G3V9H8:RET_RAT | 0.85 | 0.95 | 0.96 | 0.99 | 1.0 | 1.0 |
| sp:P35546:RET_MOUSE | 0.83 | 0.95 | 0.95 | 0.99 | 1.0 | 1.0 |
| tr:Q7JQD5:Q7JQD5_DROME | 0.17 | 0.39 | 0.55 | 0.83 | 0.93 | 0.98 |
| tr:Q7KA88:Q7KA88_DROME | 0.2 | 0.6 | 0.55 | 0.83 | 0.93 | 0.98 |
| tr:Q7KT06:Q7KT06_DROME | 0.2 | 0.6 | 0.55 | 0.83 | 0.93 | 0.98 |
| tr:Q9BHA9:Q9BHA9_DROME | 0.2 | 0.6 | 0.55 | 0.83 | 0.93 | 0.98 |

**Ret (FBgn0011829) associated phenotypes**

decreased cell death, heat sensitive, lethal - all die before end of pupal stage, partially lethal - majority die, size defective, somatic clone

(Information from FlyBase)

**Heat shock 70 kDa protein 1A**

Best gene implication in ageing for this target family came from gene O97125 annotated in UniProt release 2014_02. Annotation GO 8340 (determination of adult lifespan) was Inferred from Mutant Phenotype

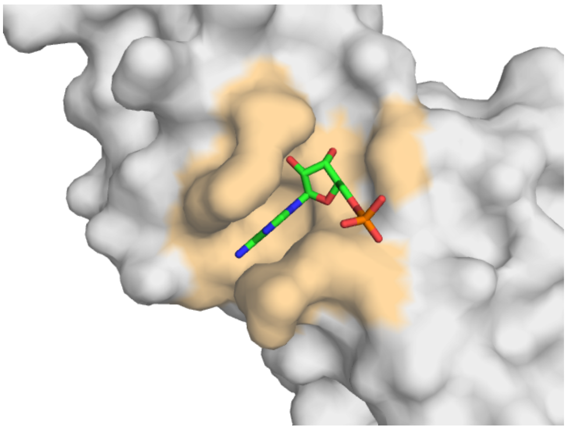

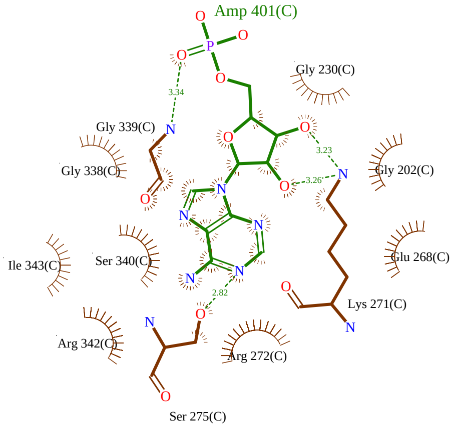

| protein | amino acids contacts (binding site) |
|---|---|
| PDB:1xqs:chainC:P08107 | G G E K R S G G S R I |
| tr:E9PN89:E9PN89_HUMAN | G G E K R S G G S R I |
| tr:E7EP94:E7EP94_HUMAN | G G E K R S G G S R I |
| tr:Q96IS6:Q96IS6_HUMAN | G G E K R S G G S R I |
| tr:E9PKE3:E9PKE3_HUMAN | G G E K R S G G S R I |
| sp:P08107:HSP71_HUMAN | G G E K R S G G S R I |
| tr:A8K5I0:A8K5I0_HUMAN | G G E K R S G G S R I |
| sp:P11142:HSP7C_HUMAN | G G E K R S G G S R I |
| sp:Q07439:HSP71_RAT | G G E K R S G G S R I |
| sp:Q61696:HS71A_MOUSE | G G E K R S G G S R I |
| sp:P17879:HS71B_MOUSE | G G E K R S G G S R I |
| tr:Q3TU85:Q3TU85_MOUSE | G G E K R S G G S R I |
| tr:Q8I0E9:Q8I0E9_DROME | G G E K R S G G S R I |
| sp:O97125:HSP68_DROME | G G E K R S G G S R I |
| sp:P29843:HSP7A_DROME | G G E K R S G G S R I |
| tr:F3YDI6:F3YDI6_DROME | G G E K R S G G S R I |
| sp:P82910:HSP70_DROME | G G E K R S G G S R I |
| sp:P02825:HSP71_DROME | G G E K R S G G S R I |
| tr:C7LA76:C7LA76_DROME | G G E K R S G G S R I |
| sp:P29844:HSP7C_DROME | G G E K R S G G S R I |
| tr:F3YDH0:F3YDH0_DROME | G G E K R S G G S R I |
| tr:C7LA75:C7LA75_DROME | G G E K R S G G S R I |
| sp:P11147:HSP7D_DROME | G G E K R S G G S R I |
| tr:F3YDH5:F3YDH5_DROME | G G E K R S G G S R I |
| tr:Q8IH88:Q8IH88_DROME | G G E K R S G G S R I |
| sp:P09446:HSP7A_CAEEL | G G E K R S G G S R I |
| sp:P10592:HSP72_YEAST | G G E K R S G G S R I |

| | whole protein | | domain-based | | contact-based | |
|---|---|---|---|---|---|---|
| protein | ident | simil | ident | simil | ident | simil |
| PDB:1xqs:chainC:P08107 | 1.0 | 1.0 | 1.0 | 1.0 | 1.0 | 1.0 |
| tr:E9PN89:E9PN89_HUMAN | 0.43 | 0.47 | 0.74 | 0.8 | 1.0 | 1.0 |
| tr:E7EP94:E7EP94_HUMAN | 0.86 | 0.86 | 1.0 | 1.0 | 1.0 | 1.0 |
| tr:Q96IS6:Q96IS6_HUMAN | 0.77 | 0.86 | 0.89 | 0.97 | 1.0 | 1.0 |
| tr:E9PKE3:E9PKE3_HUMAN | 0.83 | 0.92 | 0.89 | 0.97 | 1.0 | 1.0 |
| sp:P08107:HSP71_HUMAN | 1.0 | 1.0 | 1.0 | 1.0 | 1.0 | 1.0 |
| tr:A8K5I0:A8K5I0_HUMAN | 1.0 | 1.0 | 1.0 | 1.0 | 1.0 | 1.0 |
| sp:P11142:HSP7C_HUMAN | 0.85 | 0.95 | 0.89 | 0.97 | 1.0 | 1.0 |
| sp:Q07439:HSP71_RAT | 0.97 | 0.99 | 0.98 | 0.99 | 1.0 | 1.0 |
| sp:Q61696:HS71A_MOUSE | 0.95 | 0.99 | 0.97 | 0.99 | 1.0 | 1.0 |
| sp:P17879:HS71B_MOUSE | 0.95 | 0.99 | 0.97 | 0.99 | 1.0 | 1.0 |
| tr:Q3TU85:Q3TU85_MOUSE | 0.95 | 0.99 | 0.97 | 0.99 | 1.0 | 1.0 |
| tr:Q8I0E9:Q8I0E9_DROME | 0.68 | 0.81 | 0.84 | 0.96 | 1.0 | 1.0 |
| sp:O97125:HSP68_DROME | 0.72 | 0.9 | 0.78 | 0.93 | 1.0 | 1.0 |
| sp:P29843:HSP7A_DROME | 0.79 | 0.94 | 0.84 | 0.96 | 1.0 | 1.0 |
| tr:F3YDI6:F3YDI6_DROME | 0.74 | 0.91 | 0.77 | 0.93 | 1.0 | 1.0 |
| sp:P82910:HSP70_DROME | 0.74 | 0.91 | 0.77 | 0.93 | 1.0 | 1.0 |
| sp:P02825:HSP71_DROME | 0.74 | 0.91 | 0.77 | 0.93 | 1.0 | 1.0 |
| tr:C7LA76:C7LA76_DROME | 0.74 | 0.91 | 0.77 | 0.93 | 1.0 | 1.0 |
| sp:P29844:HSP7C_DROME | 0.58 | 0.83 | 0.59 | 0.88 | 1.0 | 1.0 |
| tr:F3YDH0:F3YDH0_DROME | 0.58 | 0.83 | 0.59 | 0.88 | 1.0 | 1.0 |
| tr:C7LA75:C7LA75_DROME | 0.81 | 0.94 | 0.85 | 0.96 | 1.0 | 1.0 |
| sp:P11147:HSP7D_DROME | 0.81 | 0.94 | 0.85 | 0.96 | 1.0 | 1.0 |
| tr:F3YDH5:F3YDH5_DROME | 0.76 | 0.91 | 0.84 | 0.96 | 1.0 | 1.0 |
| tr:Q8IH88:Q8IH88_DROME | 0.74 | 0.89 | 0.84 | 0.96 | 1.0 | 1.0 |
| sp:P09446:HSP7A_CAEEL | 0.82 | 0.94 | 0.83 | 0.96 | 1.0 | 1.0 |
| sp:P10592:HSP72_YEAST | 0.74 | 0.91 | 0.8 | 0.95 | 1.0 | 1.0 |

**Hsc70-1 (FBgn0001216) associated phenotypes**

partially lethal - majority die, some die during pupal stage

(Information from FlyBase)

**Hsp68 (FBgn0001230) associated phenotypes**
chemical resistant, long lived, neuroanatomy defective, partially lethal - majority die, some die during pupal stage, starvation stress response defective

(Information from FlyBase)

**Hsc70-1 (UniProt:P29843) annotation**
**Developmental stage**: Heat shock cognate proteins are expressed constitutively during normal development.

(Information from UniProt)

**Hsp70Aa (UniProt:P82910) annotation**
**Induction**: Heat shock induces the synthesis of seven proteins at five otherwise inactive sites in the polytene chromosomes of fruit fly larvae. Two separate sites, producing two and three copies, respectively, code for the 70 kDa protein.
**Miscellaneous**: There are two copies of the gene coding for this protein at chromosome locus 87A7.

(Information from UniProt)

**Hsp70Ab (UniProt:P02825) annotation**
**Induction**: Heat shock induces the synthesis of seven proteins at five otherwise inactive sites in the polytene chromosomes of fruit fly larvae. Two separate sites, producing two and three copies, respectively, code for the 70 kDa protein.
**Miscellaneous**: There are two copies of the gene coding for this protein at chromosome locus 87A7.

(Information from UniProt)

**Hsc70-3 (FBgn0001218) associated phenotypes**
dominant, neuroanatomy defective, partially lethal - majority die

(Information from FlyBase)

**Hsc70-3 (UniProt:P29844) annotation**
**Function**: Probably plays a role in facilitating the assembly of multimeric protein complexes inside the ER.
**Subcellular location**: Endoplasmic reticulum lumen.

(Information from UniProt)

**Hsc70-4 (UniProt:P11147) annotation**
**Subcellular location**: Cytoplasm, perinuclear region. Nucleus. Note=Localized to a meshwork of cytoplasmic fibers around the nucleus. Translocates to the nucleus after thermal stress.
**Developmental stage**: Heat shock cognate proteins are expressed constitutively during normal development.

(Information from UniProt)

**hsp-1 (WBGene00002005) associated phenotypes**
lethal, sterile

(Information from WormBase)

### 5'-AMP-activated protein kinase subunit gamma

Best gene implication in ageing for this target family came from gene P12904 annotated in UniProt release 2014_02. Annotation GO subterm of 7568 (aging) was Inferred from Mutant Phenotype

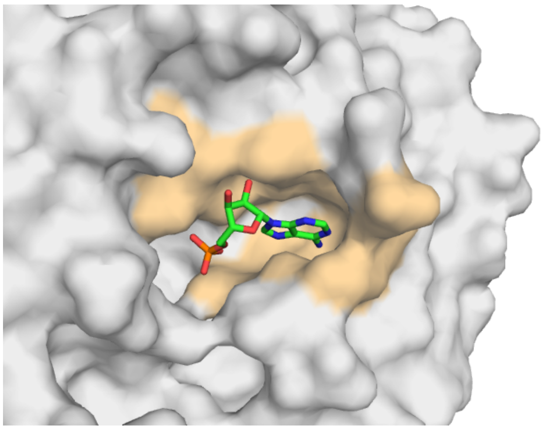

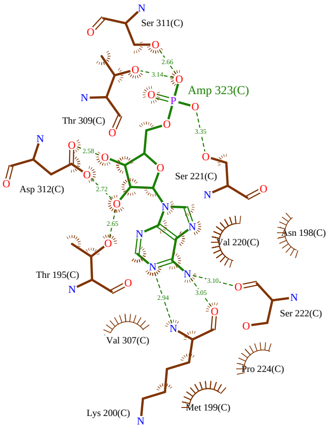


| protein | amino acids contacts (binding site) | | | | | | | | | | | |
|---|---|---|---|---|---|---|---|---|---|---|---|---|
| PDB:3tdh:chainC:P12904 | T | N | M | K | V | S | S | P | V | T | S | D |
| tr:F8W9D1:F8W9D1_HUMAN | T | N | I | A | V | S | A | P | I | S | S | D |
| tr:F8VYY9:F8VYY9_HUMAN | T | N | I | A | V | S | A | P | I | S | S | D |
| tr:B4DT70:B4DT70_HUMAN | T | D | L | A | V | S | A | P | V | S | S | D |
| sp:P54619:AAKG1_HUMAN | T | N | I | A | V | S | A | P | I | S | S | D |
| sp:Q9UGI9:AAKG3_HUMAN | T | D | L | A | V | S | A | P | V | S | S | D |
| sp:P80385:AAKG1_RAT | T | N | I | A | V | S | A | P | I | S | S | D |
| tr:D4A7E4:D4A7E4_RAT | T | D | L | A | V | S | A | P | V | S | S | D |
| sp:O54950:AAKG1_MOUSE | T | N | I | A | V | S | A | P | I | S | S | D |
| tr:Q3TWR3:Q3TWR3_MOUSE | T | N | I | A | V | S | A | P | I | S | S | D |
| sp:Q8BGM7:AAKG3_MOUSE | T | D | L | A | V | S | A | P | V | S | S | D |
| tr:Q7KNS9:Q7KNS9_DROME | T | N | I | E | V | S | A | P | I | S | S | D |
| tr:Q8IGX0:Q8IGX0_DROME | T | N | I | E | V | S | A | P | I | S | S | D |
| tr:Q0KI34:Q0KI34_DROME | T | N | I | E | V | S | A | P | I | S | S | D |
| tr:A1A703:A1A703_DROME | T | N | I | E | V | S | A | P | I | S | S | D |
| tr:A1A6Y3:A1A6Y3_DROME | T | N | I | E | V | S | A | P | I | S | S | D |
| tr:O96613:O96613_DROME | T | N | I | E | V | S | A | P | I | S | S | D |
| tr:Q8I0K6:Q8I0K6_DROME | T | N | I | E | V | S | A | P | I | S | S | D |
| tr:Q8I0A1:Q8I0A1_DROME | T | N | I | E | V | S | A | P | I | S | S | D |
| tr:Q8SXT8:Q8SXT8_DROME | T | N | I | E | V | S | A | P | I | S | S | D |
| tr:Q8I0B2:Q8I0B2_DROME | T | N | I | E | V | S | A | P | I | S | S | D |
| tr:Q7JQX9:Q7JQX9_DROME | T | N | I | E | V | S | A | P | I | S | S | D |
| tr:Q9VDD2:Q9VDD2_DROME | T | N | I | E | V | S | A | P | I | S | S | D |
| tr:Q9BHL6:Q9BHL6_CAEEL | A | D | I | L | V | S | A | P | V | S | S | D |
| sp:P12904:AAKG_YEAST | T | N | M | K | V | S | S | P | V | T | S | D |
| tr:F4ZZV2:F4ZZV2_YEASX | T | N | M | K | V | S | S | P | V | T | S | D |

| protein | whole protein ident | simil | domain-based ident | simil | contact-based ident | simil |
|---|---|---|---|---|---|---|
| PDB:3tdh:chainC:P12904 | 1.0 | 1.0 | 1.0 | 1.0 | 1.0 | 1.0 |
| tr:F8W9D1:F8W9D1_HUMAN | 0.23 | 0.56 | 0.38 | 0.81 | 0.58 | 0.77 |
| tr:F8VYY9:F8VYY9_HUMAN | 0.28 | 0.63 | 0.38 | 0.81 | 0.58 | 0.77 |
| tr:B4DT70:B4DT70_HUMAN | 0.3 | 0.69 | 0.35 | 0.8 | 0.58 | 0.77 |
| sp:P54619:AAKG1_HUMAN | 0.3 | 0.71 | 0.38 | 0.81 | 0.58 | 0.77 |
| sp:Q9UGI9:AAKG3_HUMAN | 0.2 | 0.49 | 0.35 | 0.8 | 0.58 | 0.77 |
| sp:P80385:AAKG1_RAT | 0.3 | 0.72 | 0.38 | 0.81 | 0.58 | 0.77 |
| tr:D4A7E4:D4A7E4_RAT | 0.2 | 0.49 | 0.35 | 0.8 | 0.58 | 0.77 |
| sp:O54950:AAKG1_MOUSE | 0.3 | 0.72 | 0.38 | 0.81 | 0.58 | 0.77 |
| tr:Q3TWR3:Q3TWR3_MOUSE | 0.3 | 0.72 | 0.38 | 0.81 | 0.58 | 0.77 |
| sp:Q8BGM7:AAKG3_MOUSE | 0.2 | 0.49 | 0.36 | 0.8 | 0.58 | 0.77 |
| tr:Q7KNS9:Q7KNS9_DROME | 0.21 | 0.47 | 0.38 | 0.8 | 0.58 | 0.8 |
| tr:Q8IGX0:Q8IGX0_DROME | 0.19 | 0.45 | 0.38 | 0.8 | 0.58 | 0.8 |
| tr:Q0KI34:Q0KI34_DROME | 0.2 | 0.45 | 0.38 | 0.8 | 0.58 | 0.8 |
| tr:A1A703:A1A703_DROME | 0.17 | 0.39 | 0.38 | 0.8 | 0.58 | 0.8 |
| tr:A1A6Y3:A1A6Y3_DROME | 0.17 | 0.39 | 0.38 | 0.8 | 0.58 | 0.8 |
| tr:O96613:O96613_DROME | 0.16 | 0.37 | 0.38 | 0.8 | 0.58 | 0.8 |
| tr:Q8I0K6:Q8I0K6_DROME | 0.15 | 0.34 | 0.38 | 0.8 | 0.58 | 0.8 |
| tr:Q8I0A1:Q8I0A1_DROME | 0.13 | 0.3 | 0.38 | 0.8 | 0.58 | 0.8 |
| tr:Q8SXT8:Q8SXT8_DROME | 0.12 | 0.27 | 0.38 | 0.8 | 0.58 | 0.8 |
| tr:Q8I0B2:Q8I0B2_DROME | 0.12 | 0.27 | 0.38 | 0.8 | 0.58 | 0.8 |
| tr:Q7JQX9:Q7JQX9_DROME | 0.11 | 0.26 | 0.38 | 0.8 | 0.58 | 0.8 |
| tr:Q9VDD2:Q9VDD2_DROME | 0.08 | 0.17 | 0.38 | 0.8 | 0.58 | 0.8 |
| tr:Q9BHL6:Q9BHL6_CAEEL | 0.17 | 0.41 | 0.4 | 0.81 | 0.5 | 0.72 |
| sp:P12904:AAKG_YEAST | 1.0 | 1.0 | 1.0 | 1.0 | 1.0 | 1.0 |
| tr:F4ZZV2:F4ZZV2_YEASX | 1.0 | 1.0 | 1.0 | 1.0 | 1.0 | 1.0 |

**SNF4Agamma (FBgn0264357) associated phenotypes**

feeding behavior defective, hyperactive, neuroanatomy defective, nutrition conditional, phototaxis defective, short lived, starvation stress response defective

(Information from FlyBase)

**Mitogen-activated protein kinase 10**

Best gene implication in ageing for this target family came from gene Q8WQG9 annotated in UniProt release 2014_02. Annotation GO 8340 (determination of adult lifespan) was Inferred from Mutant Phenotype

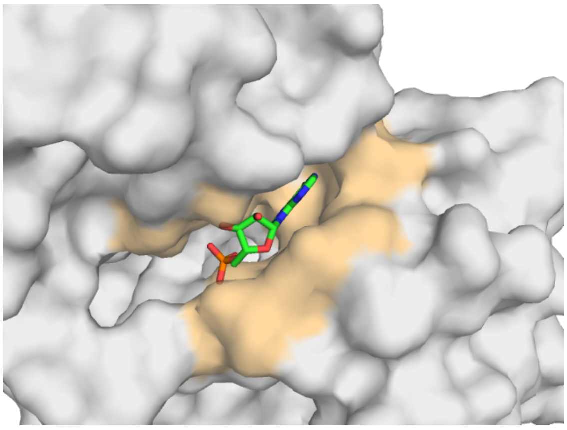

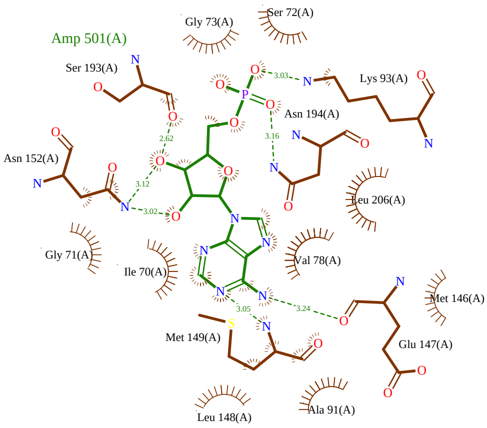

| protein | amino acids contacts (binding site) | | | | | | | | | | | | | | |
|---|---|---|---|---|---|---|---|---|---|---|---|---|---|---|---|
| PDB:4kke:chainA:P53779 | I | G | S | G | V | A | K | M | E | L | M | N | S | N | L |
| tr:A6NF29:A6NF29_HUMAN | I | G | S | G | V | A | K | M | E | L | M | N | S | N | L |
| tr:D7R525:D7R525_HUMAN | I | G | S | G | V | A | K | M | E | L | M | N | S | N | L |
| tr:J3KNK1:J3KNK1_HUMAN | I | G | S | G | V | A | K | M | E | L | M | N | S | N | L |
| sp:P45983:MK08_HUMAN | I | G | S | G | V | A | K | M | E | L | M | N | S | N | L |
| sp:P45984:MK09_HUMAN | I | G | S | G | V | A | K | M | E | L | M | N | S | N | L |
| tr:A1L4K2:A1L4K2_HUMAN | I | G | S | G | V | A | K | M | E | L | M | N | S | N | L |
| sp:P53779:MK10_HUMAN | I | G | S | G | V | A | K | M | E | L | M | N | S | N | L |
| tr:F1LP66:F1LP66_RAT | I | G | S | G | V | A | K | M | E | L | M | N | S | N | L |
| tr:D4A5V8:D4A5V8_RAT | I | G | S | G | V | A | K | M | E | L | M | N | S | N | L |
| sp:P49186:MK09_RAT | I | G | S | G | V | A | K | M | E | L | M | N | S | N | L |
| tr:B0VXR6:B0VXR6_RAT | I | G | S | G | V | A | K | M | E | L | M | N | S | N | L |
| tr:D3ZQ33:D3ZQ33_RAT | I | G | S | G | V | A | K | M | E | L | M | N | S | N | L |
| tr:D3Z1Z4:D3Z1Z4_MOUSE | I | G | S | G | V | A | K | M | E | L | M | N | S | N | L |
| tr:A6P3E4:A6P3E4_MOUSE | I | G | S | G | V | A | K | M | E | L | M | N | S | N | L |
| sp:Q91Y86:MK08_MOUSE | I | G | S | G | V | A | K | M | E | L | M | N | S | N | L |
| tr:Q544A0:Q544A0_MOUSE | I | G | S | G | V | A | K | M | E | L | M | N | S | N | L |
| tr:Q3TQZ7:Q3TQZ7_MOUSE | I | G | S | G | V | A | K | M | E | L | M | N | S | N | L |
| tr:Q80W82:Q80W82_MOUSE | I | G | S | G | V | A | K | M | E | L | M | N | S | N | L |
| tr:Q7TSJ7:Q7TSJ7_MOUSE | I | G | S | G | V | A | K | M | E | L | M | N | S | N | L |
| tr:Q78GB8:Q78GB8_MOUSE | I | G | S | G | V | A | K | M | E | L | M | N | S | N | L |
| sp:Q9WTU6:MK09_MOUSE | I | G | S | G | V | A | K | M | E | L | M | N | S | N | L |
| tr:G3X8U9:G3X8U9_MOUSE | I | G | S | G | V | A | K | M | E | L | M | N | S | N | L |
| tr:Q5NCK8:Q5NCK8_MOUSE | I | G | S | G | V | A | K | M | E | L | M | N | S | N | L |
| tr:Q80W80:Q80W80_MOUSE | I | G | S | G | V | A | K | M | E | L | M | N | S | N | L |
| tr:Q8C9D4:Q8C9D4_MOUSE | I | G | S | G | V | A | K | M | E | L | M | N | S | N | L |
| tr:E9QN59:E9QN59_MOUSE | I | G | S | G | V | A | K | M | E | L | M | N | S | N | L |
| tr:E1JHD6:E1JHD6_DROME | I | G | S | G | V | A | K | M | E | L | M | N | S | N | L |
| sp:P92208:JNK_DROME | I | G | S | G | V | A | K | M | E | L | M | N | S | N | L |
| sp:Q8WQG9:JNK1_CAEEL | I | G | S | G | V | A | K | M | E | L | M | N | S | N | L |
| sp:P32485:HOG1_YEAST | V | G | M | G | V | A | K | T | E | L | Q | D | S | N | C |

| protein | whole protein ident | simil | domain-based ident | simil | contact-based ident | simil |
|---|---|---|---|---|---|---|
| PDB:4kke:chainA:P53779 | 1.0 | 1.0 | 1.0 | 1.0 | 1.0 | 1.0 |
| tr:A6NF29:A6NF29_HUMAN | 0.43 | 0.46 | 0.69 | 0.73 | 1.0 | 1.0 |
| tr:D7R525:D7R525_HUMAN | 0.41 | 0.44 | 0.66 | 0.7 | 1.0 | 1.0 |
| tr:J3KNK1:J3KNK1_HUMAN | 0.63 | 0.7 | 0.76 | 0.8 | 1.0 | 1.0 |
| sp:P45983:MK08_HUMAN | 0.83 | 0.89 | 0.96 | 0.99 | 1.0 | 1.0 |
| sp:P45984:MK09_HUMAN | 0.76 | 0.86 | 0.91 | 0.97 | 1.0 | 1.0 |
| tr:A1L4K2:A1L4K2_HUMAN | 0.82 | 0.89 | 0.94 | 0.99 | 1.0 | 1.0 |
| sp:P53779:MK10_HUMAN | 1.0 | 1.0 | 1.0 | 1.0 | 1.0 | 1.0 |
| tr:F1LP66:F1LP66_RAT | 0.76 | 0.84 | 0.89 | 0.96 | 1.0 | 1.0 |
| tr:D4A5V8:D4A5V8_RAT | 0.77 | 0.86 | 0.93 | 0.98 | 1.0 | 1.0 |
| sp:P49186:MK09_RAT | 0.76 | 0.86 | 0.91 | 0.97 | 1.0 | 1.0 |
| tr:B0VXR6:B0VXR6_RAT | 0.92 | 0.92 | 1.0 | 1.0 | 1.0 | 1.0 |
| tr:D3ZQ33:D3ZQ33_RAT | 0.99 | 1.0 | 1.0 | 1.0 | 1.0 | 1.0 |
| tr:D3Z1Z4:D3Z1Z4_MOUSE | 0.61 | 0.65 | 0.78 | 0.81 | 1.0 | 1.0 |
| tr:A6P3E4:A6P3E4_MOUSE | 0.74 | 0.8 | 0.94 | 0.99 | 1.0 | 1.0 |
| sp:Q91Y86:MK08_MOUSE | 0.75 | 0.81 | 0.96 | 0.99 | 1.0 | 1.0 |
| tr:Q544A0:Q544A0_MOUSE | 0.75 | 0.81 | 0.96 | 0.99 | 1.0 | 1.0 |
| tr:Q3TQZ7:Q3TQZ7_MOUSE | 0.89 | 0.9 | 1.0 | 1.0 | 1.0 | 1.0 |
| tr:Q80W82:Q80W82_MOUSE | 0.89 | 0.9 | 1.0 | 1.0 | 1.0 | 1.0 |
| tr:Q7TSJ7:Q7TSJ7_MOUSE | 0.83 | 0.89 | 0.96 | 0.99 | 1.0 | 1.0 |
| tr:Q78GB8:Q78GB8_MOUSE | 0.92 | 0.92 | 1.0 | 1.0 | 1.0 | 1.0 |
| sp:Q9WTU6:MK09_MOUSE | 0.77 | 0.86 | 0.93 | 0.98 | 1.0 | 1.0 |
| tr:G3X8U9:G3X8U9_MOUSE | 0.82 | 0.89 | 0.94 | 0.99 | 1.0 | 1.0 |
| tr:Q5NCK8:Q5NCK8_MOUSE | 0.76 | 0.86 | 0.91 | 0.97 | 1.0 | 1.0 |
| tr:Q80W80:Q80W80_MOUSE | 0.98 | 0.99 | 0.97 | 0.99 | 1.0 | 1.0 |
| tr:Q8C9D4:Q8C9D4_MOUSE | 1.0 | 1.0 | 1.0 | 1.0 | 1.0 | 1.0 |
| tr:E9QN59:E9QN59_MOUSE | 0.94 | 0.94 | 1.0 | 1.0 | 1.0 | 1.0 |
| tr:E1JHD6:E1JHD6_DROME | 0.62 | 0.74 | 0.84 | 0.95 | 1.0 | 1.0 |
| sp:P92208:JNK_DROME | 0.62 | 0.74 | 0.84 | 0.95 | 1.0 | 1.0 |
| sp:Q8WQG9:JNK1_CAEEL | 0.45 | 0.6 | 0.8 | 0.94 | 1.0 | 1.0 |
| sp:P32485:HOG1_YEAST | 0.29 | 0.64 | 0.47 | 0.81 | 0.6 | 0.62 |

**bsk (FBgn0000229) associated phenotypes**

RU486 conditional, cell death defective, chemical resistant, chemical sensitive, conditional, decreased cell growth, dominant, heat sensitive, increased cell death, long lived, neuroanatomy defective, neurophysiology defective, nutrition conditional, oxidative stress response defective, planar polarity defective, progressive, short lived, somatic clone, starvation stress response defective, stress response defective, wound healing defective

(Information from FlyBase)

**bsk (UniProt:P92208) annotation**

**Function**: Responds to activation by environmental stress by phosphorylating a number of transcription factors, primarily components of AP-1 such as Jra and also the transcriptional repressor aop, and thus regulates transcriptional activity. Component of the immune response activated by bacterial infection, and is involved in wound healing and in dorsal closure, a morphogenetic movement during embryogenesis. Controls the expression of a phosphatase, puckered, at the edges of wounded epidermal tissue and in the dorsal epithelium during dorsal closure. (PubMed:10433922, PubMed:11784101, PubMed:8946915, PubMed:9224720).

**Cofactor**:Mg(2+)

**Enzyme regulation**: Activated by threonine and tyrosine phosphorylation by the dual specificity kinase, hep. Inhibited by dual specificity phosphatase, puckered. (PubMed:10433922).

**Subcellular location**: Cytoplasm

**Tissue specificity**: During gastrulation, expression is seen in cells undergoing morphogenetic movements. By stage 9 of embryonic development, expression is ubiquitous. At stages 12-14, expression occurs in epidermis and central nervous system. At stage 15, expression is restricted to ventral nerve cord, brain and some peripheral neurons. In larvae, expression is seen in all imaginal disks, with highest levels in wing and eye disks, and in the CNS. Adults express the protein in fat body and hemocytes. (PubMed:8946915, PubMed:8946916).

**Developmental stage**: Expressed maternally and zygotically through to adult (male and female). (PubMed:8946915).
**Domain**: The TXY motif contains the threonine and tyrosine residues whose phosphorylation activates the MAP kinases.
**Ptm**: Dually phosphorylated on Thr-181 and Tyr-183, which activates the enzyme.

(Information from UniProt)

### jnk-1 (WBGene00002178) associated phenotypes

aldicarb hypersensitive, pathogen resistance increased, protein phosphorylation reduced, shortened life span, thermotolerance reduced

(Information from WormBase)

### jnk-1 (UniProt:Q8WQG9) annotation

**Function**: Responds to activation by environmental stress by phosphorylating a number of transcription factors, and thus regulates transcriptional activity (By similarity). May coordinate locomotion by acting via type-D GABAergic motor neurons and regulate synaptic vesicle transport in conjunction with unc-16. (, PubMed:10393177, PubMed:11738026).
**Cofactor**:Mg(2+) Evidence=(PubMed:10393177);
**Enzyme regulation**: Activated by threonine and tyrosine phosphorylation by either of the dual specificity kinases, jkk-1 and sek-1. (PubMed:10393177).
**Subunit**: Binds to the scaffolding protein, unc-16. Unc-16 also binds other components of the JNK signaling pathway. (PubMed:11738026).
**Domain**: The TXY motif contains the threonine and tyrosine residues whose phosphorylation activates the MAP kinases.
**Ptm**: Dually phosphorylated on Thr-276 and Tyr-278, which activates the enzyme.

(Information from UniProt)

## 5'-AMP-activated protein kinase subunit gamma-1

Best gene implication in ageing for this target family came from gene P12904 annotated in UniProt release 2014_02. Annotation GO subterm of 7568 (aging) was Inferred from Mutant Phenotype

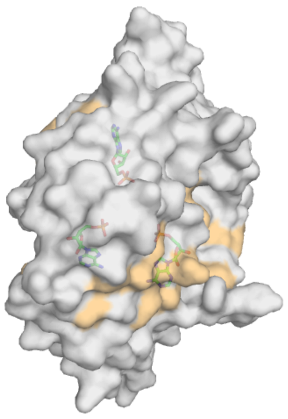

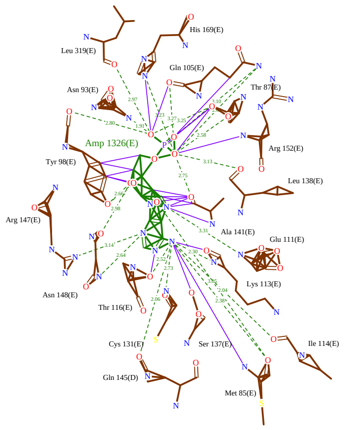

| protein | amino acids contacts (binding site) |
|---|---|
| PDB:2uv4:chainA:P54619 | - - - - - - - - - - - - - - - - - - - - - - - - - - - - - - - - - - L T N I A |
| PDB:2uv6:chainA:P54619 | - - - - - - - - - - - - - - - - - - - - - - - - - - - - - - - - - - L T N I A |
| PDB:2uv7:chainA:P54619 | - - - - - - - - - - - - - - - - - - - - - - - - - - - - - - - - - - L T N I A |
| PDB:4cfe:chainE:P54619 | R M T T D N L H Y L V Q Y E E H K I T C S L F A V R N I H R H K L K L T N I A |
| PDB:4cff:chainE:P54619 | R M T T D N L H Y L V Q Y E E H K I T C S L F A V R N I H R H K L K L T N I A |
| tr:F8VYY9:F8VYY9_HUMAN | R M T T D N L H Y L V Q Y E E H K I T C S L F A V R N I H R H K L K L T N I A |
| sp:P54619:AAKG1_HUMAN | R M T T D N L H Y L V Q Y E E H K I T C S L F A V R N I H R H K L K L T N I A |
| sp:P80385:AAKG1_RAT | R M T T D N L H Y L V Q Y E E H K I T C S L F A V R N I H R H K L K L T N I A |
| sp:O54950:AAKG1_MOUSE | R M T T D N L H Y L V Q Y E E H K I T C S L F A V R N I H R H K L K L T N I A |
| tr:Q3TWR3:Q3TWR3_MOUSE | R M T T D N L H Y L V Q Y E E H K I T C S L F A V R N I H R H K L K L T N I A |
| tr:Q7KNS9:Q7KNS9_DROME | R M T T D K L Q Y N A S E Q E H K L T S S L Y A I H S I H R H K L F L T N I E |
| tr:Q0KI34:Q0KI34_DROME | R M T T D K L Q Y N A S E Q E H K L T S S L Y A I H S I H R H K L F L T N I E |
| tr:A1A703:A1A703_DROME | R M T T D K L Q Y N A S E Q E H K L T S S L Y A I H S I H R H K L F L T N I E |
| tr:A1A6Y3:A1A6Y3_DROME | R M T T D K L Q Y N A S E Q E H K L T S S L Y A I H S I H R H K L F L T N I E |
| tr:O96613:O96613_DROME | R M T T D K L Q Y N A S E Q E H K L T S S L Y A I H S I H R H K L F L T N I E |
| tr:Q8I0K6:Q8I0K6_DROME | R M T T D K L Q Y N A S E Q E H K L T S S L Y A I H S I H R H K L F L T N I E |
| tr:Q8I0A1:Q8I0A1_DROME | R M T T D K L Q Y N A S E Q E H K L T S S L Y A I H S I H R H K L F L T N I E |
| tr:Q8SXT8:Q8SXT8_DROME | R M T T D K L Q Y N A S E Q E H K L T S S L Y A I H S I H R H K L F L T N I E |
| tr:Q8I0B2:Q8I0B2_DROME | R M T T D K L Q Y N A S E Q E H K L T S S L Y A I H S I H R H K L F L T N I E |
| tr:Q7JQX9:Q7JQX9_DROME | R M T T D K L Q Y N A S E Q E H K L T S S L Y A I H S I H R H K L F L T N I E |
| tr:Q9VDD2:Q9VDD2_DROME | R M T T D K L Q Y N A S E Q E H K L T S S L Y A I H S I H R H K L F L T N I E |
| tr:Q9BHL6:Q9BHL6_CAEEL | R M T T D K L C H S E R R A D Q Q I H Y S L H A V E S V H R H K M S L A D I L |
| sp:P12904:AAKG_YEAST | V L T T D N I Q Y - - - E L K L Q L G S P L F A C E S S G R Q Y L A L T N M K |
| tr:F4ZZV2:F4ZZV2_YEASX | V L T T D N I Q Y - - - E L K L Q L G S P L F A C E S S G R Q Y L A L T N M K |

| | whole protein | | domain-based | | contact-based | |
|---|---|---|---|---|---|---|
| protein | ident | simil | ident | simil | ident | simil |
| PDB:2uv4:chainA:P54619 | 0.99 | 1.0 | 1.0 | 1.0 | 0.59 | 0.17 |
| PDB:2uv6:chainA:P54619 | 0.99 | 1.0 | 0.99 | 1.0 | 0.57 | 0.17 |
| PDB:2uv7:chainA:P54619 | 0.99 | 0.99 | 0.99 | 1.0 | 0.57 | 0.16 |
| PDB:4cfe:chainE:P54619 | 1.0 | 1.0 | 1.0 | 1.0 | 1.0 | 1.0 |
| PDB:4cff:chainE:P54619 | 1.0 | 1.0 | 1.0 | 1.0 | 1.0 | 1.0 |
| tr:F8VYY9:F8VYY9_HUMAN | 0.84 | 0.84 | 0.86 | 0.87 | 1.0 | 1.0 |
| sp:P54619:AAKG1_HUMAN | 1.0 | 1.0 | 1.0 | 1.0 | 1.0 | 1.0 |
| sp:P80385:AAKG1_RAT | 0.96 | 0.99 | 0.98 | 1.0 | 1.0 | 1.0 |
| sp:O54950:AAKG1_MOUSE | 0.97 | 0.99 | 0.99 | 1.0 | 1.0 | 1.0 |
| tr:Q3TWR3:Q3TWR3_MOUSE | 0.97 | 0.99 | 0.99 | 1.0 | 1.0 | 1.0 |
| tr:Q7KNS9:Q7KNS9_DROME | 0.39 | 0.54 | 0.64 | 0.87 | 0.63 | 0.78 |
| tr:Q0KI34:Q0KI34_DROME | 0.37 | 0.53 | 0.64 | 0.88 | 0.63 | 0.78 |
| tr:A1A703:A1A703_DROME | 0.33 | 0.46 | 0.64 | 0.89 | 0.63 | 0.78 |
| tr:A1A6Y3:A1A6Y3_DROME | 0.32 | 0.46 | 0.64 | 0.88 | 0.63 | 0.78 |
| tr:O96613:O96613_DROME | 0.31 | 0.44 | 0.64 | 0.89 | 0.63 | 0.78 |
| tr:Q8I0K6:Q8I0K6_DROME | 0.28 | 0.4 | 0.64 | 0.89 | 0.63 | 0.78 |
| tr:Q8I0A1:Q8I0A1_DROME | 0.25 | 0.35 | 0.64 | 0.89 | 0.63 | 0.78 |
| tr:Q8SXT8:Q8SXT8_DROME | 0.22 | 0.31 | 0.64 | 0.89 | 0.63 | 0.78 |
| tr:Q8I0B2:Q8I0B2_DROME | 0.22 | 0.31 | 0.64 | 0.89 | 0.63 | 0.78 |
| tr:Q7JQX9:Q7JQX9_DROME | 0.21 | 0.3 | 0.64 | 0.89 | 0.63 | 0.78 |
| tr:Q9VDD2:Q9VDD2_DROME | 0.14 | 0.2 | 0.64 | 0.89 | 0.63 | 0.78 |
| tr:Q9BHL6:Q9BHL6_CAEEL | 0.29 | 0.47 | 0.54 | 0.84 | 0.48 | 0.63 |
| sp:P12904:AAKG_YEAST | 0.3 | 0.71 | 0.32 | 0.75 | 0.37 | 0.57 |
| tr:F4ZZV2:F4ZZV2_YEASX | 0.3 | 0.71 | 0.32 | 0.75 | 0.37 | 0.57 |

**SNF4Agamma (FBgn0264357) associated phenotypes**

feeding behavior defective, hyperactive, neuroanatomy defective, nutrition conditional, phototaxis defective, short lived, starvation stress response defective

(Information from FlyBase)

## 5'-AMP-activated protein kinase subunit gamma-1

Best gene implication in ageing for this target family came from gene P12904 annotated in UniProt release 2014_02. Annotation GO subterm of 7568 (aging) was Inferred from Mutant Phenotype

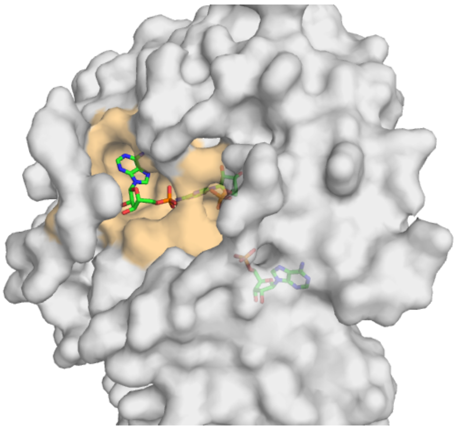

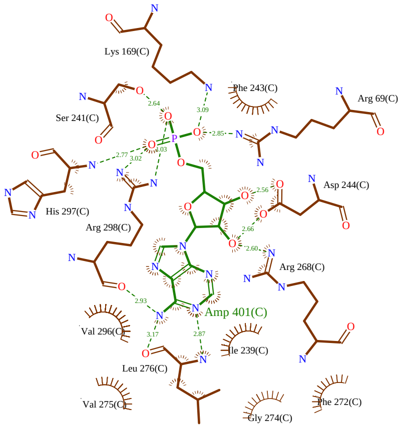


| protein | amino acids contacts (binding site) |
|---|---|
| PDB:2v8q:chainE:P80385 | R M T T D Q K P L V I H R P K T N I A V S A P I S F D R F G V L V H R V I S S |
| PDB:2v92:chainE:P80385 | R M T T D Q K P L V I H R P K T N I A V S A P I S F D R F G V L V H R V I S S |
| PDB:2v9j:chainE:P80385 | R M T T D Q K P L V I H R P K T N I A V S A P I S F D R F G V L V H R V I S S |
| PDB:2y8l:chainE:P80385 | R M T T D Q K P L V I H R P K T N I A V S A P I S F D R F G V L V H R V I S S |
| PDB:2y8q:chainE:P80385 | R M T T D Q K P L V I H R P K T N I A V S A P I S F D R F G V L V H R V I S S |
| PDB:2ya3:chainE:P80385 | R M T T D Q K P L V I H R P K T N I A V S A P I S F D R F G V L V H R V I S S |
| PDB:4cfh:chainE:P80385 | R M T T D Q K P L V I H R P K T N I A V S A P I S F D R F G V L V H R V I S S |
| PDB:4eai:chainC:P80385 | R M T T D - K P L V I H R P K T N I A V S A P I S F D R F G V L V H R V I S S |
| PDB:4eaj:chainC:P80385 | R M T T D - K P L V I H R P K T N I A V S A P I S F D R F G V L V H R V I S S |
| PDB:4eal:chainC:P80385 | R M T T D Q K P L V I H R P K T N I A V S A P I S F D R - - V L V H R V I S S |
| tr:F8VYY9:F8VYY9_HUMAN | R M T T D Q K P L V I H R P K T N I A V S A P I S F D R F G V L V H R V I S S |
| sp:P54619:AAKG1_HUMAN | R M T T D Q K P L V I H R P K T N I A V S A P I S F D R F G V L V H R V I S S |
| sp:P80385:AAKG1_RAT | R M T T D Q K P L V I H R P K T N I A V S A P I S F D R F G V L V H R V I S S |
| sp:O54950:AAKG1_MOUSE | R M T T D Q K P L V I H R P K T N I A V S A P I S F D R F G V L V H R V I S S |
| tr:Q3TWR3:Q3TWR3_MOUSE | R M T T D Q K P L V I H R P K T N I A V S A P I S F D R F G V L V H R V I S S |
| tr:Q7KNS9:Q7KNS9_DROME | R M T T D H M P L V I H R P K T N I E V S A P I A F D R F G V Q V H R V I S S |
| tr:Q8IGX0:Q8IGX0_DROME | R M T T D H M P L V I H R P K T N I E V S A P I A F D R F G V Q V H R V I S S |
| tr:Q0KI34:Q0KI34_DROME | R M T T D H M P L V I H R P K T N I E V S A P I A F D R F G V Q V H R V I S S |
| tr:A1A703:A1A703_DROME | R M T T D H M P L V I H R P K T N I E V S A P I A F D R F G V Q V H R V I S S |
| tr:A1A6Y3:A1A6Y3_DROME | R M T T D H M P L V I H R P K T N I E V S A P I A F D R F G V Q V H R V I S S |
| tr:O96613:O96613_DROME | R M T T D H M P L V I H R P K T N I E V S A P I A F D R F G V Q V H R V I S S |
| tr:Q8I0K6:Q8I0K6_DROME | R M T T D H M P L V I H R P K T N I E V S A P I A F D R F G V Q V H R V I S S |
| tr:Q8I0A1:Q8I0A1_DROME | R M T T D H M P L V I H R P K T N I E V S A P I A F D R F G V Q V H R V I S S |
| tr:Q8SXT8:Q8SXT8_DROME | R M T T D H M P L V I H R P K T N I E V S A P I A F D R F G V Q V H R V I S S |
| tr:Q8I0B2:Q8I0B2_DROME | R M T T D H M P L V I H R P K T N I E V S A P I A F D R F G V Q V H R V I S S |
| tr:Q7JQX9:Q7JQX9_DROME | R M T T D H M P L V I H R P K T N I E V S A P I A F D R F G V Q V H R V I S S |
| tr:Q9VDD2:Q9VDD2_DROME | R M T T D H M P L V I H R P K T N I E V S A P I A F D R F G V Q V H R V I S S |
| tr:Q9BHL6:Q9BHL6_CAEEL | R M T T D D R P F V V H R P K A D I L V S A P I A F D R F G V Q V H R I V S S |
| sp:P12904:AAKG_YEAST | V L T T D D L D T A S G R P Y T N M K V S S P V E Y D R F G V Y V H R F V T S |
| tr:F4ZZV2:F4ZZV2_YEASX | V L T T D D L D T A S G R P Y T N M K V S S P V E Y D R F G V Y V H R F V T S |

| protein | whole protein ident | simil | domain-based ident | simil | contact-based ident | simil |
|---|---|---|---|---|---|---|
| PDB:2v8q:chainE:P80385 | 1.0 | 1.0 | 1.0 | 1.0 | 1.0 | 1.0 |
| PDB:2v92:chainE:P80385 | 1.0 | 1.0 | 1.0 | 1.0 | 1.0 | 1.0 |
| PDB:2v9j:chainE:P80385 | 1.0 | 1.0 | 1.0 | 1.0 | 1.0 | 1.0 |
| PDB:2y8l:chainE:P80385 | 1.0 | 1.0 | 1.0 | 1.0 | 1.0 | 1.0 |
| PDB:2y8q:chainE:P80385 | 1.0 | 1.0 | 1.0 | 1.0 | 1.0 | 1.0 |
| PDB:2ya3:chainE:P80385 | 1.0 | 1.0 | 1.0 | 1.0 | 1.0 | 1.0 |
| PDB:4cfh:chainE:P80385 | 1.0 | 1.0 | 1.0 | 1.0 | 1.0 | 1.0 |
| PDB:4eai:chainC:P80385 | 1.0 | 1.0 | 1.0 | 1.0 | 0.97 | 0.95 |
| PDB:4eaj:chainC:P80385 | 1.0 | 1.0 | 1.0 | 1.0 | 0.97 | 0.95 |
| PDB:4eal:chainC:P80385 | 1.0 | 1.0 | 1.0 | 1.0 | 0.95 | 0.9 |
| tr:F8VYY9:F8VYY9_HUMAN | 0.83 | 0.84 | 0.86 | 0.87 | 1.0 | 1.0 |
| sp:P54619:AAKG1_HUMAN | 0.96 | 0.99 | 0.98 | 1.0 | 1.0 | 1.0 |
| sp:P80385:AAKG1_RAT | 1.0 | 1.0 | 1.0 | 1.0 | 1.0 | 1.0 |
| sp:O54950:AAKG1_MOUSE | 0.98 | 0.99 | 0.98 | 0.99 | 1.0 | 1.0 |
| tr:Q3TWR3:Q3TWR3_MOUSE | 0.98 | 0.99 | 0.98 | 0.99 | 1.0 | 1.0 |
| tr:Q7KNS9:Q7KNS9_DROME | 0.39 | 0.54 | 0.64 | 0.87 | 0.88 | 0.9 |
| tr:Q8IGX0:Q8IGX0_DROME | 0.36 | 0.53 | 0.63 | 0.88 | 0.88 | 0.9 |
| tr:Q0KI34:Q0KI34_DROME | 0.37 | 0.53 | 0.63 | 0.88 | 0.88 | 0.9 |
| tr:A1A703:A1A703_DROME | 0.32 | 0.46 | 0.64 | 0.88 | 0.88 | 0.9 |
| tr:A1A6Y3:A1A6Y3_DROME | 0.32 | 0.46 | 0.64 | 0.88 | 0.88 | 0.9 |
| tr:O96613:O96613_DROME | 0.31 | 0.44 | 0.64 | 0.88 | 0.88 | 0.9 |
| tr:Q8I0K6:Q8I0K6_DROME | 0.28 | 0.4 | 0.64 | 0.88 | 0.88 | 0.9 |
| tr:Q8I0A1:Q8I0A1_DROME | 0.24 | 0.35 | 0.64 | 0.88 | 0.88 | 0.9 |
| tr:Q8SXT8:Q8SXT8_DROME | 0.22 | 0.31 | 0.64 | 0.88 | 0.88 | 0.9 |
| tr:Q8I0B2:Q8I0B2_DROME | 0.22 | 0.31 | 0.64 | 0.88 | 0.88 | 0.9 |
| tr:Q7JQX9:Q7JQX9_DROME | 0.21 | 0.3 | 0.64 | 0.88 | 0.88 | 0.9 |
| tr:Q9VDD2:Q9VDD2_DROME | 0.14 | 0.2 | 0.64 | 0.88 | 0.88 | 0.9 |
| tr:Q9BHL6:Q9BHL6_CAEEL | 0.29 | 0.47 | 0.54 | 0.83 | 0.72 | 0.86 |
| sp:P12904:AAKG_YEAST | 0.29 | 0.67 | 0.33 | 0.75 | 0.5 | 0.68 |
| tr:F4ZZV2:F4ZZV2_YEASX | 0.29 | 0.67 | 0.33 | 0.75 | 0.5 | 0.68 |

**SNF4Agamma (FBgn0264357) associated phenotypes**

feeding behavior defective, hyperactive, neuroanatomy defective, nutrition conditional, phototaxis defective, short lived, starvation stress response defective

(Information from FlyBase)

## Aprataxin

Best gene implication in ageing for this target family came from gene Q08702 via mapping the annotation from Ensembl YOR258W via mapping the annotation from EntrezGene 854432 via mapping the annotation from GenAgeModels 1072 annotated in GenAge release 17.

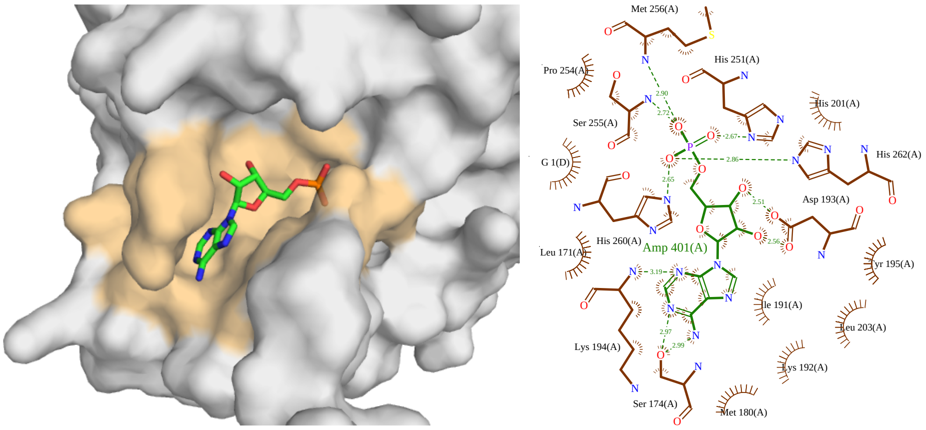

| protein | amino acids contacts (binding site) |
|---|---|
| PDB:4ndf:chainA:Q7Z2E3 | G L S M I K D K Y K H L H P S M H H |
| PDB:4ndh:chainA:Q7Z2E3 | G L S M I K D K Y K H L H P S M H H |
| PDB:4ndi:chainA:Q7Z2E3 | G L S M I K D K Y Q H L H P S M H H |
| tr:F5HRF8:F5HRF8_HUMAN | G L S M I K D K Y K H L H P S M H H |
| sp:Q7Z2E3:APTX_HUMAN | G L S M I K D K Y K H L H P S M H H |
| sp:Q8K4H4:APTX_RAT | G L S M I K D K Y K H L H P S M H H |
| sp:Q7TQC5:APTX_MOUSE | G L S M I K D K Y K H L H P S M H H |
| sp:Q8MSG8:APTX_DROME | A L D N I A D K F K H L H P S M H H |
| sp:Q08702:APTX_YEAST | A L Y T I R D S F K H L H P S M H H |

| protein | whole protein ident | whole protein simil | domain-based ident | domain-based simil | contact-based ident | contact-based simil |
|---|---|---|---|---|---|---|
| PDB:4ndf:chainA:Q7Z2E3 | 0.98 | 0.99 | 1.0 | 1.0 | 1.0 | 1.0 |
| PDB:4ndh:chainA:Q7Z2E3 | 0.98 | 0.99 | 1.0 | 1.0 | 1.0 | 1.0 |
| PDB:4ndi:chainA:Q7Z2E3 | 0.97 | 0.99 | 0.99 | 1.0 | 0.94 | 0.97 |
| tr:F5HRF8:F5HRF8_HUMAN | 0.81 | 0.81 | 1.0 | 1.0 | 1.0 | 1.0 |
| sp:Q7Z2E3:APTX_HUMAN | 1.0 | 1.0 | 1.0 | 1.0 | 1.0 | 1.0 |
| sp:Q8K4H4:APTX_RAT | 0.78 | 0.88 | 0.91 | 0.97 | 1.0 | 1.0 |
| sp:Q7TQC5:APTX_MOUSE | 0.82 | 0.91 | 0.91 | 0.97 | 1.0 | 1.0 |
| sp:Q8MSG8:APTX_DROME | 0.09 | 0.17 | 0.41 | 0.76 | 0.72 | 0.8 |
| sp:Q08702:APTX_YEAST | 0.14 | 0.31 | 0.23 | 0.54 | 0.67 | 0.77 |

**CG5316 (UniProt:Q8MSG8) annotation**

**Function**: DNA-binding protein involved in single-strand DNA break repair, double-strand DNA break repair and base excision repair. Resolves abortive DNA ligation intermediates formed either at base excision sites, or when DNA ligases attempt to repair non- ligatable breaks induced by reactive oxygen species. Catalyzes the release of adenylate groups covalently linked to 5'-phosphate termini, resulting in the production of 5'-phosphate termini that can be efficiently rejoined (By similarity).

**Subcellular location**: Nucleus

**Domain**: The HIT domain is required for enzymatic activity.

**Domain**: The C2H2-type zinc finger mediates DNA-binding.

(Information from UniProt)
